# Mice Lacking Alternatively Activated (M2) Macrophages Show Impairments in Restorative Sleep after Sleep Loss and in Cold Environment

**DOI:** 10.1038/s41598-018-26758-x

**Published:** 2018-06-05

**Authors:** Ashley Massie, Erin Boland, Levente Kapás, Éva Szentirmai

**Affiliations:** 1 Elson S. Floyd College of Medicine, Department of Biomedical Sciences, Washington State University, Spokane, Washington USA; 2 Sleep and Performance Research Center, Washington State University, Spokane, Washington USA

## Abstract

The relationship between sleep, metabolism and immune functions has been described, but the cellular components of the interaction are incompletely identified. We previously reported that systemic macrophage depletion results in sleep impairment after sleep loss and in cold environment. These findings point to the role of macrophage-derived signals in maintaining normal sleep. Macrophages exist either in resting form, classically activated, pro-inflammatory (M1) or alternatively activated, anti-inflammatory (M2) phenotypes. In the present study we determined the contribution of M2 macrophages to sleep signaling by using IL-4 receptor α-chain-deficient [IL-4Rα knockout (KO)] mice, which are unable to produce M2 macrophages. Sleep deprivation induced robust increases in non-rapid-eye-movement sleep (NREMS) and slow-wave activity in wild-type (WT) animals. NREMS rebound after sleep deprivation was ~50% less in IL-4Rα KO mice. Cold exposure induced reductions in rapid-eye-movement sleep (REMS) and NREMS in both WT and KO mice. These differences were augmented in IL-4Rα KO mice, which lost ~100% more NREMS and ~25% more REMS compared to WTs. Our finding that M2 macrophage-deficient mice have the same sleep phenotype as mice with global macrophage depletion reconfirms the significance of macrophages in sleep regulation and suggests that the main contributors are the alternatively activated M2 cells.

## Introduction

The most widely accepted sleep model describes sleep regulation as the outcome of the interactions between homeostatic and circadian processes^1^. The circadian process is linked to the function of the suprachiasmatic nucleus of the hypothalamus. The mechanisms and the substrate for the homeostatic sleep regulation are not fully understood, but most studies place these mechanisms within the brain, such as the basal forebrain, where adenosine signaling is thought to play a role^2^. Increasing number of studies suggest, however, that in addition to the circadian and homeostatic factors, other processes, which are linked to metabolism and the immune system, are also critical in sleep regulation^3,4^. Furthermore, a significant component of metabolic and immune sleep signaling arises from outside of the central nervous system. To develop a more comprehensive view of sleep regulation, we need to understand both the role of brain mechanisms and the role of peripheral immune and metabolic signaling in sleep.

Sleep-promoting immune signaling in pro-inflammatory conditions has been described^3^. Systemic bacterial or viral infections, viral and bacterial cell wall components all induce sleep^5–8^. It is assumed that macrophages play a key role in sleep responses to inflammatory challenges caused by microbes^9–11^. In response to pro-inflammatory stimuli, macrophages become “classically activated” M1 cells and produce large quantities of nitric oxide (NO) and pro-inflammatory cytokines such as tumor necrosis factor alpha (TNFα) and interleukin-1 beta (IL-1β)^12^. Pro-inflammatory cytokines and NO stimulate non-rapid eye movement sleep (NREMS) and likely contribute to sleep responses in inflammatory conditions, as well^13–15^.

We recently demonstrated that macrophages also play a role in sleep signaling in non-inflammatory conditions. Macrophage depletion by clodronate-containing liposomes (CCL) suppresses rebound sleep responses after sleep loss in mice^16^. Further, macrophage-depleted animals cannot maintain normal sleep amounts when exposed to moderately cold temperatures. These findings show that in the absence of an inflammatory challenge, under two physiological conditions (after short-term sleep loss and at 10 °C ambient temperature), macrophage function/signaling is required for maintaining normal sleep.

Resting macrophages can go through one of two distinct activation programs. Classic activation of macrophages results in the formation of pro-inflammatory M1 cells in response to type 1 T helper (Th1) cytokines, such as interferon gamma (IFNγ). M1 macrophages are characterized by increased antigen presenting capacity, increased synthesis of pro-inflammatory cytokines and toxic mediators such as NO, and augmented complement-mediated phagocytosis^12^. Alternative activation of macrophages leads to the M2 polarization of the cells. M2 macrophages do not produce pro-inflammatory cytokines and NO, but they contribute to defense against extracellular parasites, promote wound healing and are associated with allergic responses. Alternative activation occurs in response to IL-4 and IL-13 acting on Type 1 and Type 2 IL-4 receptors. Signaling through these receptors is dependent on the IL-4Rα subunit, which is the signal-transducing component of both receptor complexes^17^. Genetic ablation of the IL-4Rα subunit prevents the M2 polarization of macrophages in response to a broad range of alternative activation-inducing stimuli, while classical activation of macrophages remains intact or even enhanced^18–21^. Due to the inability of macrophages to go through alternative activation in IL-4Rα KO mice, this genetic model is widely used for studying the contribution of M2 macrophages in various physiological and pathological processes (e.g.^22–24^).

In the present study, we investigated the contribution of M2 macrophages in sleep regulation by using IL-4Rα knockout (KO) mice. Our main finding is that selective M2 deficiency reproduces the sleep phenotype observed after the generalized macrophage depletion by CCL, which affects resting, M1 and M2 macrophages. M2 macrophage deficiency leads to the inability of the animals to maintain normal amounts of recovery sleep after sleep loss and normal amounts of sleep in a mildly cold environment. This is consistent with the hypothesis that alternatively activated, M2, macrophages contribute to the immune signaling of sleep.

## Results

### Spontaneous sleep, locomotor activity, and body temperature at thermoneutral ambient temperature (30 °C)

**Figure 1:**
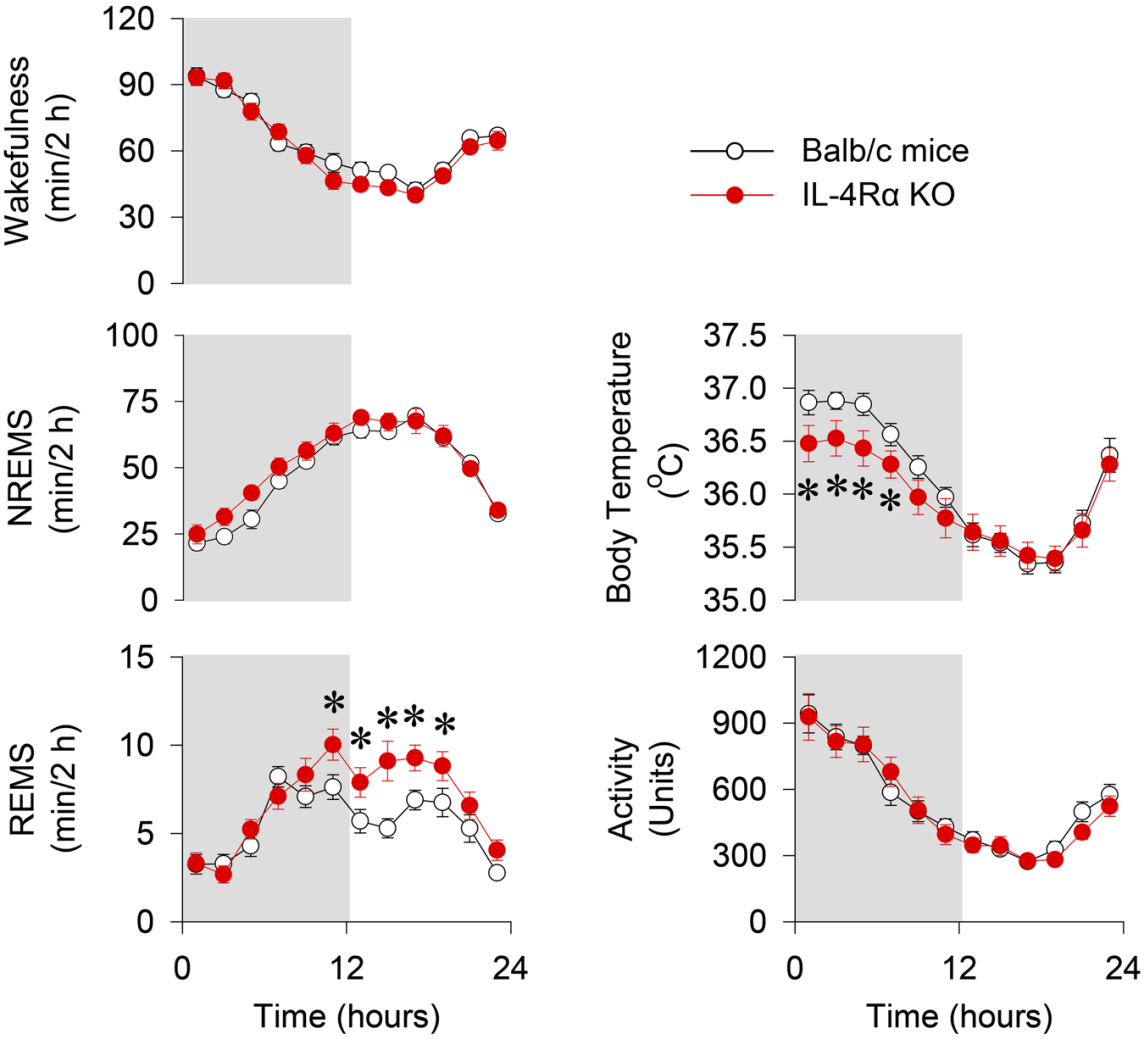
Spontaneous diurnal rhythms of wakefulness, non-rapid-eye movement sleep (NREMS), rapid-eye movement sleep (REMS), body temperature and locomotor activity in WT (white circles) and IL-4Rα KO (red circles) mice at 30 °C ambient temperature. Data are calculated in 2-h time blocks; grey shaded areas represent the dark period. Error bars represent SEM. Asterisks indicate significant difference between WT and KO mice, Tukey’s HSD test, *P* < 0.05.

Balb/c and IL-4Rα KO mice showed typical nocturnal pattern of sleep-wake activity with more NREMS and rapid-eye-movement sleep (REMS) during the light phase than during the dark phase (Fig. 1, Table 1). The amounts of wakefulness and NREMS were similar between the two genotypes. IL-4Rα KO mice had significantly more REMS during the light phase when compared to controls. This difference was due to a significantly higher number of REMS episodes observed in the KO mice when compared with control mice (25.3 ± 1.7 episodes in the Balb/c mice vs. 35.8 ± 3.1 episodes in the IL-4Rα KO mice, p < 0.05). There was no significant difference in the REMS episode durations between the two genotypes (78.2 ± 2.2 s in the Balb/c mice vs. 77.7 ± 1.8 s in IL-4Rα KO mice).

**Table 1:** Comparisons of spontaneous sleep-wake activity, body temperature and motor activity rhythms between IL-4 Rα KO and Balb/c mice – statistical results.

Spontaneous WAKE	df	F	p
Genotype	1,20	1.3	n.s.
Time	11,220	63.8	<0.05
Time x genotype	11,220	0.9	n.s.
**Spontaneous NREMS**			
Genotype	1,20	2.2	n.s.
Time	11,220	76.0	<0.05
Time x genotype	11,220	0.9	n.s.
**Spontaneous REMS**			
Genotype	1,20	5.1	<0.05
Time	11,220	25.3	<0.05
Time x genotype	11,220	2.8	<0.05
**Spontaneous Body Temperature**			
Genotype	1,20	1.0	n.s.
Time	11,220	95.8	<0.05
Time x genotype	11,220	3.0	<0.05
**Spontaneous motor activity**			
Genotype	1,20	0.1	n.s.
Time	11,220	48.7	<0.05
Time x genotype	11,220	0.5	n.s.

Body temperature and motor activity showed normal diurnal rhythms with higher body temperature and motor activity during the active dark phase and lower body temperature and motor activity during the light phase. The body temperature of KO mice was significantly lower during the dark phase as compared to controls (Fig. 1, Table 1). Locomotor activity was not significantly different between wild-type (WT) and KO animals.

### Effects of sleep deprivation on sleep-wake activity

**Figure 2:**
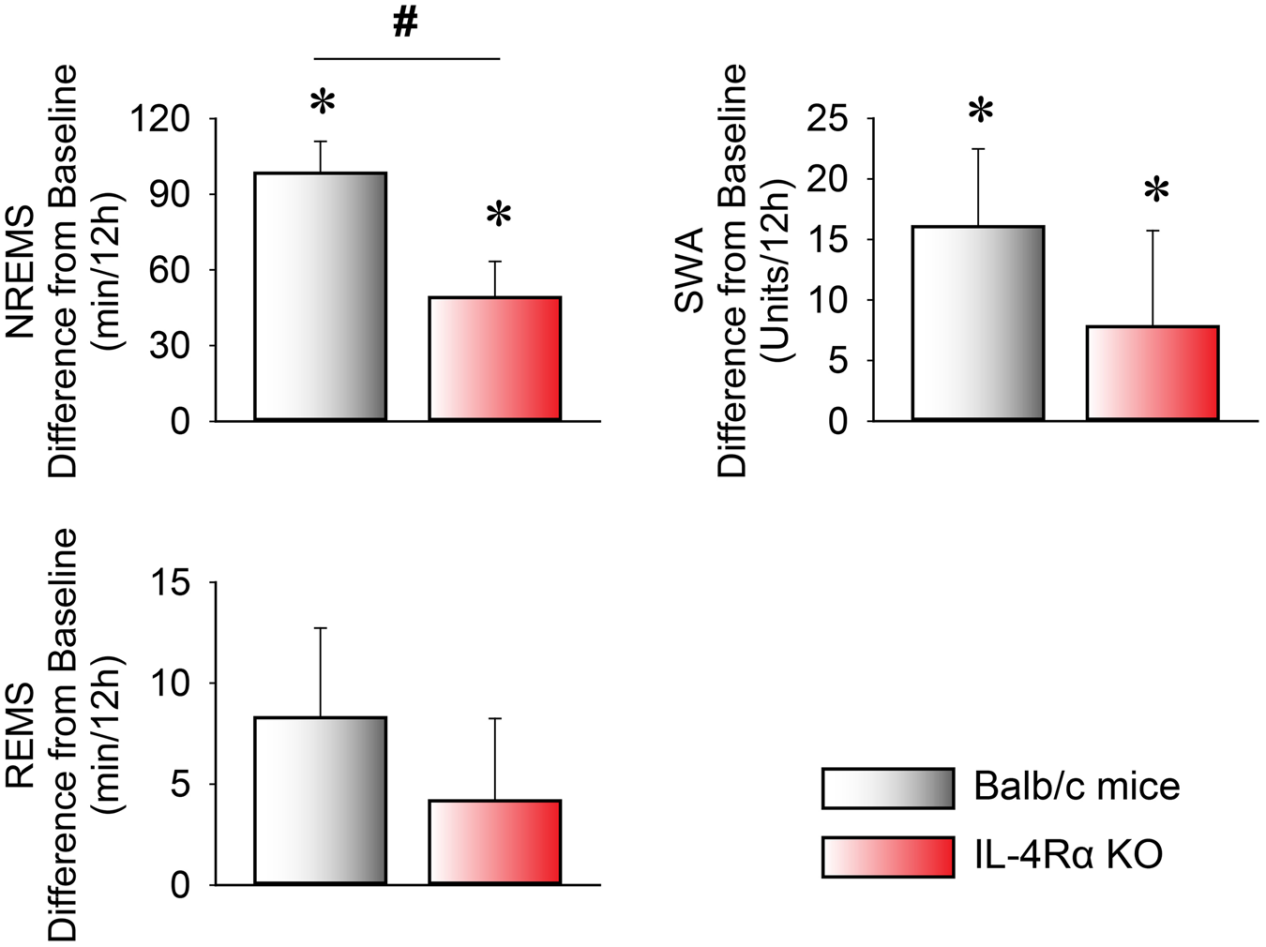
Changes in NREMS, REMS, and electroencephalographic slow wave activity (EEG SWA) during the 12-h dark period following sleep deprivation in WT (white bars) and IL-4Rα KO (red bars) mice at an ambient temperature of 30 °C. Data are calculated in 12-h time blocks, as differences from the baseline. Error bars represent SEM. ^*^Significant difference from baseline (paired *t*-test, p < 0.05), ^#^significant difference between genotypes (Student’s *t*-test, *P* < 0.05).

During six hours of sleep deprivation by gentle handling, WT and IL-4Rα KO mice lost similar amounts of sleep (sleep loss in WT: NREMS 148.9 ± 5.8 min, REMS 14.5 ± 1.3 min; KO: NREMS 145.4 ± 8.1 min, REMS 20.3 ± 2.2 min). In WT mice, sleep deprivation induced significant rebound NREMS increases of 95.3 ± 13.4 min in the following 12-h recovery period compared to baseline (baseline: 234.7 ± 12.9 min; recovery: 333 ± 12.4 min; Fig. 2, Table 2). In IL-4Rα KO mice, the rebound NREMS increase was significantly attenuated by ~ 50% (baseline: 281.3 ± 8.3 min; recovery: 330.3 ± 14.2 min; Fig. 2, Table 2).

**Table 2:** The effect of 6 h sleep deprivation on NREMS, REMS and SWA in IL-4Rα KO and Balb/c mice – statistical results.

WAKE	df	F	p
Genotype	1,16	0.6	n.s.
Treatment	1,16	62.7	<0.05
Treatment x genotype	1,16	1.2	n.s.
**NREMS**			
Genotype	1,16	2.3	n.s.
Treatment	1,16	59.6	<0.05
Treatment x genotype	1,16	6.6	<0.05
**REMS**			
Genotype	1,16	1.6	n.s.
Treatment	1,16	4.2	n.s.
Treatment x genotype	1,16	0.4	n.s.
**Body Temperature**			
Genotype	1,16	1.8	n.s.
Treatment	1,16	5.1	<0.05
Treatment x genotype	1,16	0.0	n.s.
**Motor activity**			
Genotype	1,16	3.4	n.s.
Treatment	1,16	5.4	<0.05
Treatment x genotype	1,16	0.2	n.s.
**SWA**			
Genotype	1,12	0.0	n.s.
Treatment	1,12	5.4	<0.05
Treatment x genotype	1,12	0.6	n.s.

In WT mice, the rebound increase in NREMS was due to the significantly higher number (78.5 ± 2.2 episodes on the baseline day vs. 87.6 ± 3.6 episodes on the recovery day, p < 0.05) and longer duration of the individual NREMS episodes (190.3 ± 9.2 s on the baseline day vs 229.8 ± 9.8 s on the recovery day, p < 0.05). The number and duration of NREMS episodes did not change significantly in response to sleep deprivation in the IL-4Rα KO mice (84.3 ± 6.1 episodes on the baseline day vs. 87.6 ± 3.7 episodes on the recovery day; 190.9 ± 14.5 s on the baseline day vs. 231.1 ± 17.8 s on the recovery day).

There was a tendency towards increased REMS after sleep deprivation in both genotypes; the responses of the WT and KO mice did not differ significantly. Electroencephalographic (EEG) slow-wave activity (SWA) was significantly increased after sleep deprivation in both WT and KO mice, but there was no significant difference between genotypes (Fig. 2, Table 2).

### Effects of cold exposure on sleep-wake activity and body temperature

**Figure 3:**
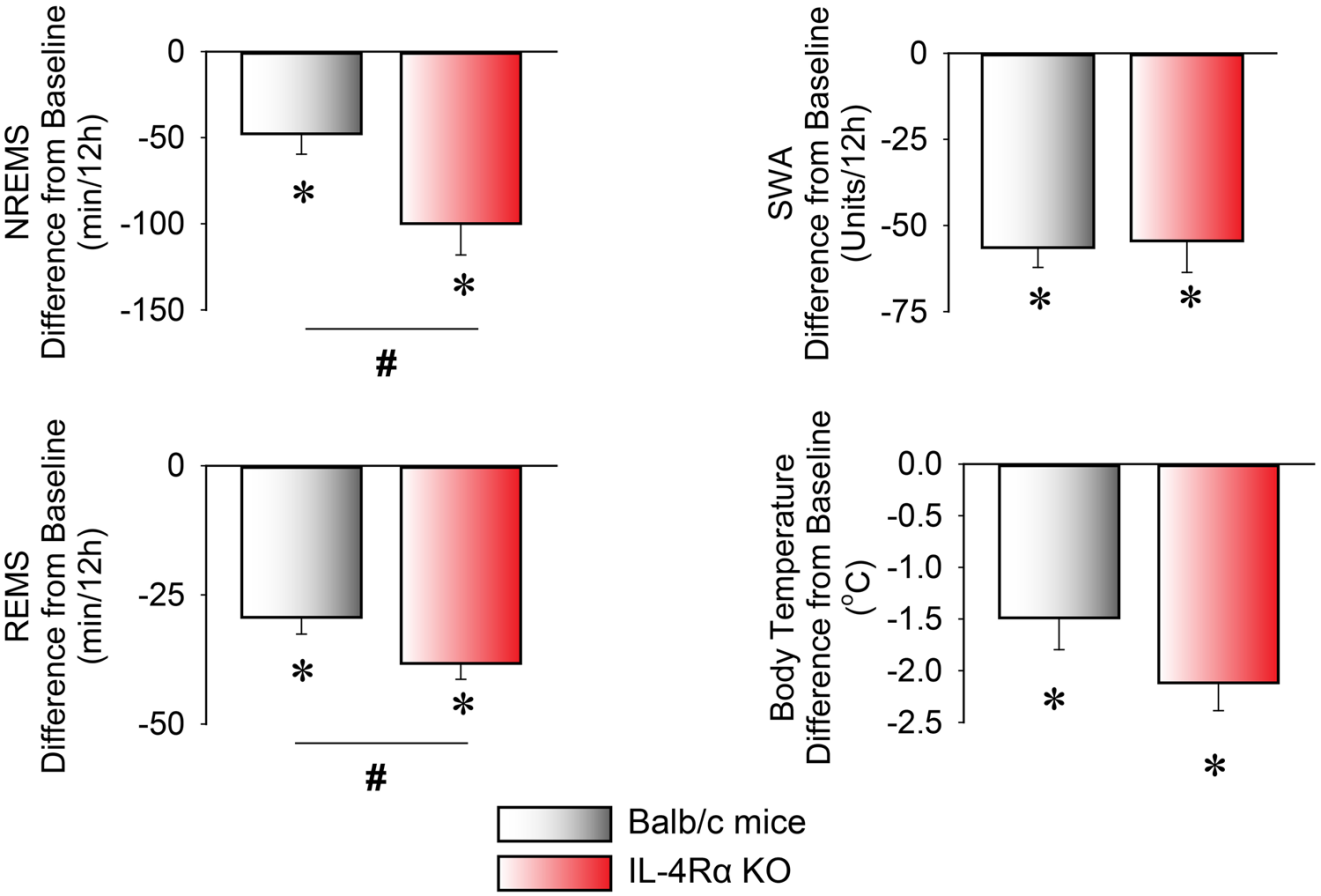
Changes in NREMS, REMS, and EEG SWA in the first 12 hours of 10 °C cold exposure in WT (white bars) and IL-4 Rα KO (red bars) mice. Data are calculated in 12-h time blocks, as differences from the baseline at 30 °C. Error bars represent SEM. ^*^Significant difference from baseline (paired *t*-test, p < 0.05), ^#^significant difference between genotypes (Student’s *t*-test, *P < *0.05).

In response to 10 °C cold exposure, the total amounts of NREMS and REMS were significantly decreased in both WT and KO mice, when compared to their respective baselines (Fig. 3, Table 3). These changes were significantly augmented in the KO mice who lost ~100% more NREMS (99.9 ± 18.2 min in IL-4Rα KO mice vs 47.7 ± 11.8 min in WT mice, p < 0.05) and ~25% more REMS (38.2 ± 3.1 min in IL-4Rα KO mice vs 29.3 ± 3.2 min in WT, p < 0.05) than control mice during the first 12 h of the cold period.

**Table 3:** The effect of cold exposure on NREMS, REMS and SWA in IL-4Rα KO and Balb/c mice – statistical results.

WAKE	df	F	p
Genotype	1,15	0.5	n.s.
Treatment	1,15	52.7	<0.05
Treatment x genotype	1,15	1.2	n.s.
**NREMS**			
Genotype	1,15	1.7	n.s.
Treatment	1,15	43.5	<0.05
Treatment x genotype	1,15	5.4	<0.05
**REMS**			
Genotype	1,15	3.0	n.s.
Treatment	1,15	228.5	<0.05
Treatment x genotype	1,15	3.9	<0.05
**Body Temperature**			
Genotype	1,16	1.8	n.s.
Treatment	1,16	5.1	<0.05
Treatment x genotype	1,16	0.0	n.s.
**SWA**			
Genotype	1,14	0.0	n.s.
Treatment	1,14	130.0	<0.05
Treatment x genotype	1,14	0.8	n.s.

Neither the number nor the average duration of individual NREMS episodes were altered in the WT mice in the cold. Suppression of REMS was due to the significantly fewer (23.7 ± 2.6 episodes on the baseline day vs. 1.38 ± 0.6 episodes on the cold exposure day, p < 0.05) and shorter (78.5 ± 3.4 s on the baseline day vs. 34.2 ± 16.8 s on the cold day, p < 0.05) REMS episodes.

In IL-4Rα KO mice, the changes in sleep were due to the significantly fewer NREMS (79.1 ± 4.1 episodes on the baseline day vs. 56.7 ± 5.0 episodes on the cold day, p < 0.05) and REMS episodes (29.8 ± 2.9 episodes on the baseline day vs. 0.5 ± 0.4 episodes on the cold day, p < 0.05) in response to the cold. The average duration of the REMS episodes on the cold day could not be assessed statistically due to the low number of the episodes. There was no significant difference in the amounts of sleep during the second half of the cold exposure between the genotypes (data not shown). EEG SWA was significantly reduced in both groups of mice without significant difference between genotypes (Table 3).

Cold exposure induced significant decreases in body temperature in both WT and IL-4 Rα KO mice during the 12-h dark period. Cold-induced hypothermia was significantly augmented IL-4 Rα KO mice compared to controls (2.3 ± 0.2 °C decrease in IL-4 Rα KO mice vs 1.5 ± 0.3 °C decrease in WTs, p < 0.05).

### Effects of cold exposure on VO_2_

**Figure 4:**
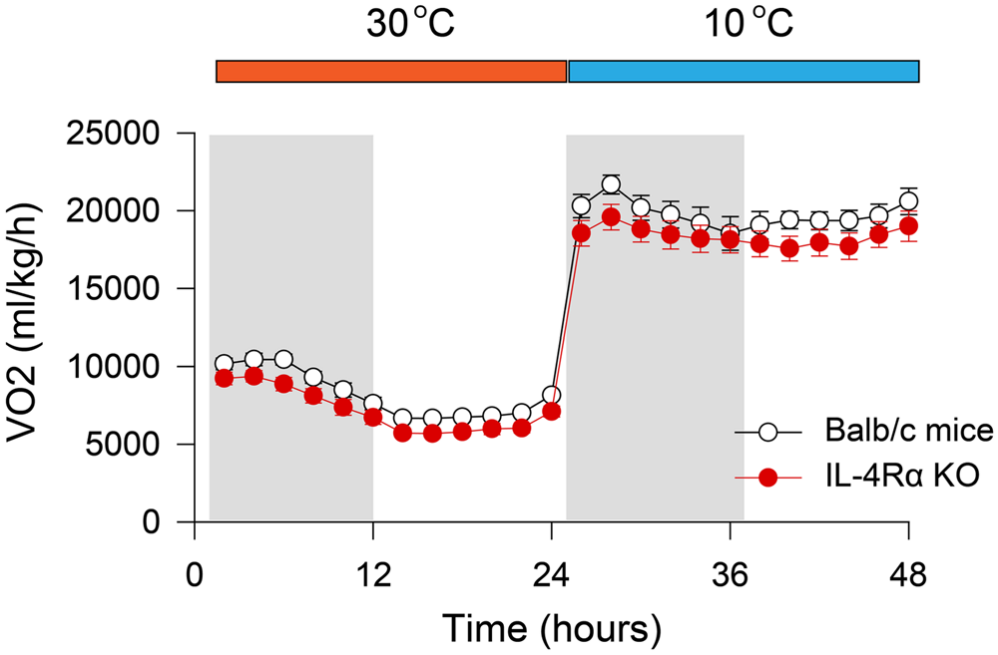
Changes in VO_2_ during the baseline day at 30 °C ambient temperature and during the 10 °C cold exposure day in WT (white symbols) and IL-4 Rα KO (red symbols) mice. Data are calculated in 2-h time blocks.

On the baseline day, clear diurnal changes were present in both groups of mice with increased VO_2_ during the dark (active) phase of the day (Fig. 4). In response to cold, VO_2_ increased 2–3-fold in both genotypes. Throughout the 48-h experimental period, VO_2_ of IL-4Rα KO mice was slightly but consistently below the values recorded in WTs, but statistically significant differences between the two genotypes were not found (3-way ANOVA; genotype effect: F(1,14) = 2.75, n.s.; ambient temperature effect: F(1,14) = 908.9, p < 0.001; time of the day effect: F(11,154) = 41.3, p < 0.001; genotype x ambient temperature interaction: F(1,14) = 0.2, n.s.).

### Brown adipose tissue (BAT) expression of IL-4 mRNA

**Figure 5:**
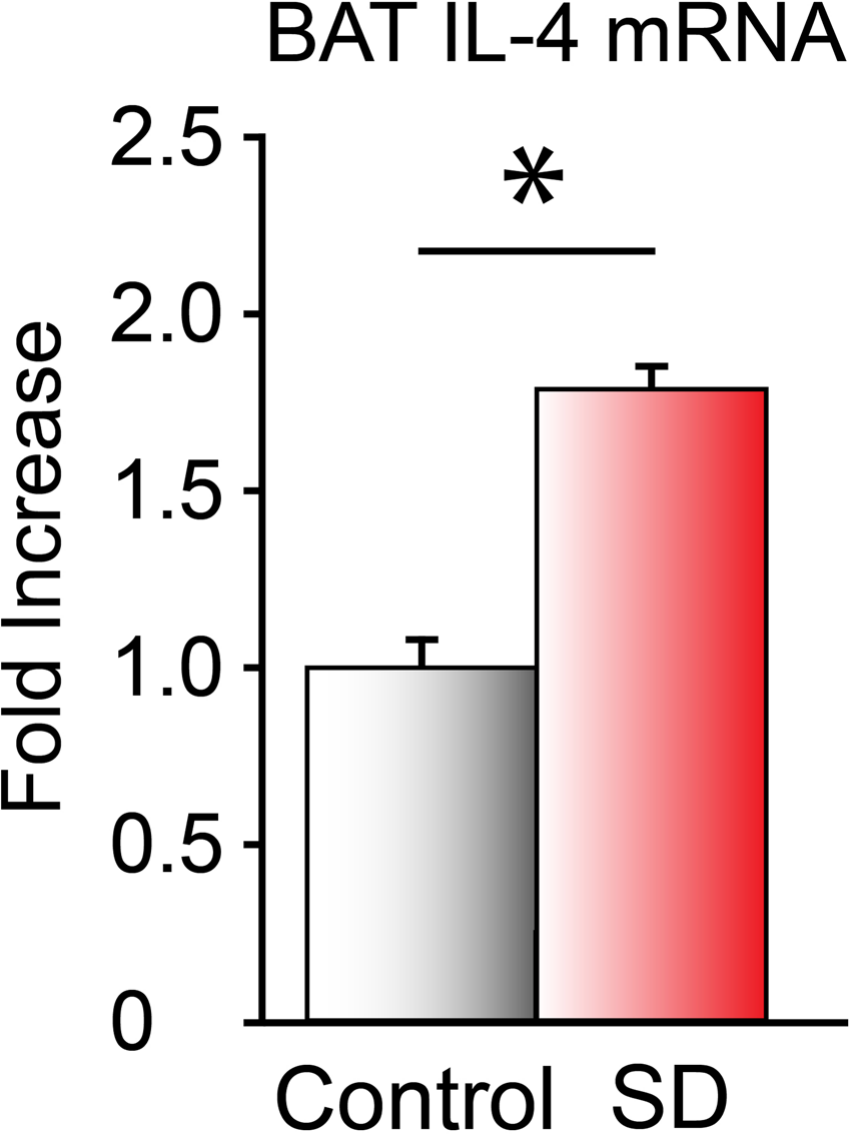
IL-4 mRNA expression in interscapular BAT in mice allowed to sleep ad libitum and in sleep deprived mice. ^*^Significant difference between groups (Student’s *t*-test, *P* < 0.05).

Six hours of sleep deprivation resulted in a ~75% increase in IL-4 mRNA expression in the BAT of WT mice (Fig. 5).

## Discussion

In the present study, we investigated the contribution of M2 macrophages to sleep regulation by using genetically modified mice, in which the pathway of alternative macrophage activation is defective. Our major finding is that mice with selective M2 deficiency have suppressed sleep rebound after sleep loss and they are unable to maintain normal sleep amounts in a mildly cold environment.

Sleep loss elicits subsequent rebound increases in sleep, presumably by the action of homeostatic sleep-regulatory mechanisms. Typically, not only the amount of sleep is enhanced after sleep deprivation, but also EEG SWA, which is often regarded as a measure of sleep pressure^1^. In our study, WT and KO mice lost about the same amount of sleep during 6 h sleep deprivation. While WT mice accumulated ~100 min extra sleep in the 12 h period following sleep deprivation accompanied by a 15% increase in SWA, in KO animals NREMS rebound was about the half of that. This points to impaired homeostatic sleep processes and suggests a role for M2 cells in compensatory increases in sleep after sleep loss. Our finding is consistent with the observations that spontaneous wakefulness and/or experimental sleep loss increases circulating monocyte number^25–27^ and favors Th2 immune responses and M2 polarization in humans. For example, insomnia unrelated to medical disorders^28^ or experimental sleep restriction^29^ shifts in the Th1/Th2 balance toward Th2 dominance. Further, overnight sleep deprivation stimulates the number of IL-10-producing monocytes^30^; IL-10 is a product and marker of M2 cells^17^. IL-10 production by peripheral mononuclear (M2) cells positively correlates with subsequent amounts of delta sleep^31^.

The ambient temperature of 30 °C is within the thermoneutral range for mice. In industrialized societies, human life is lived at thermoneutrality due to clothing and temperate housing temperatures. Performing *in vivo* experiments at thermoneutrality is a way to replicate thermal conditions experienced by humans in everyday life^32^. At 30 °C ambient temperature, there was no significant difference in the amount of wakefulness and NREMS, and in the motor activities of WT and IL-4Rα KO mice suggesting that under thermoneutral conditions IL-4 signaling mechanisms are dispensable for maintaining NREMS amounts and activity. In the KO animals, the number of REMS episodes and the overall amount of REMS were increased mainly at dark-light transition and during the light phase. Since there are mutual inhibitory interactions between NREMS intensity and REMS propensity^33^, increased REMS may be the reflection of the decreased activity of NREMS-promoting mechanisms. Alternatively, increased REMS may be due altered activity of REMS-generating mechanisms, independent of NREMS.

It is well recognized that exposure to subthermoneutral ambient temperatures decreases sleep in mice^34,35^. Exposure to 10 °C temperature elicited NREMS and REMS loss in the WT animals, suppressed EEG SWA and led to a significant drop in body temperature. Both the NREMS and REMS responses in IL-4Rα KO mice were significantly augmented; they lost more than twice the amount of NREMS as compared to WTs. They also lost significantly more REMS, which almost completely disappeared in the KO animals. Both genotypes exhibited a robust, ~60% decrease in EEG SWA. Decreased EEG SWA might indicate weak sleep pressure, but it is also possible that it is the consequence of decreased cortical activity due to the moderately decreased cortical temperature in the cold^36^.

The role of IL-4 system in maintaining body temperature has been addressed previously with partly contradicting results. In one study, IL-4 KO mice and mice with disrupted IL-4/IL-13 signaling in myeloid cells showed impaired cold tolerance as indicated by enhanced drop in body temperature in cold challenge at 4 °C^22^. In another study, IL-4Rα KO mice showed the same degree of cold tolerance as WTs^24^. In our sleep experiments, body temperature of IL-4Rα KO mice was slightly, but significantly, decreased at thermoneutral temperature during the first part of the dark phase. In the calorimetry experiment, there was no significant difference in VO_2_ between WT and KO mice suggesting that metabolic heat production is intact. To note, in the sleep experiments, 4–6-month-old mice were used, but in the calorimetry, the age of the animals was about 3 months. Three to 6-month old mice are considered mature adult mice; in this age range both the dynamic changes characteristic of the early development as well as aging-related changes are absent, thus the findings from these two experimental groups are comparable. In fact, energy expenditure of 3-month-old mice is not different from that of 5-month-old animals^37^. At 10 °C ambient temperature, IL-4Rα KO mice showed a slight tendency towards an augmented hypothermic response. These findings, together with the fact that changes in sleep architecture in the cold was more robust in the KOs, suggests that they have increased cold sensitivity, but the clarification of the role of the IL-4 signaling system in thermoregulation awaits more investigation.

Selective deficiency in M2 macrophage formation, overall, reproduced the sleep phenotype of the non-selective macrophage depletion induced by CCL^16^. In both models, homeostatic sleep response was impaired and there was a deficiency in generating NREMS in cold environment. This suggests that within the macrophage population, M2 macrophages play a key, but likely not exclusive, role in sleep signaling. Non-selective depletion of resting, M1, and M2 macrophages completely prevented homeostatic NREMS rebound after sleep loss, while selective deficiency in M2 cells led only to a 50% attenuation of the responses. This suggests that in addition to M2 cells, signaling by other macrophage population(s) may also contribute to the homeostatic regulation of sleep.

One possible mechanism through which M2 signaling may contribute to sleep regulation is through BAT thermogenesis. Alternative activation of macrophages stimulates BAT thermogenesis^22^, and BAT thermogenesis is an important metabolic signal that facilitates sleep^34,38^. Rebound sleep after sleep loss is greatly attenuated in mice with defective BAT heat production, a phenotype that is shared with macrophage-depleted and M2 cell-deficient models. Our finding that sleep deprivation upregulates IL-4 expression in BAT indicates that the key component of the molecular machinery for the alternative activation of macrophages is present in BAT and activated in response to sleep loss.

The relationship between sleep and the alternative activation of other macrophage populations is less understood, but possibly exists. Liver Kupffer cells constitute more than 80% of all tissue macrophages in the body^39^ and they go through alternative activation in response to IL-4^40^. The connection between liver and sleep has been proposed. Thermal stimulation of liver increases NREMS^41^, and the sleep-inducing effects of cafeteria diet are abolished by Kupffer cell depletion^42^. Further, antigen uptake in the liver, a measure of phagocytotic activity of macrophages, is enhanced by sleep deprivation^43^. The resident macrophages of the brain, microglia, also express IL-4Rα, and they are also capable of M2 polarization in response IL-4^44,45^. The role of microglia in sleep regulation has not been studied in depth. Theoretical models exist, in which microglia and purinergic receptors are parts of networks that mediate sleep and immune interactions (reviewed in^46^). These models will require mechanistic testing. Short-term, 1–8 h, sleep loss does not lead to the activation of microglia^47,48^. Further, NREMS rebound after sleep loss is not attenuated by minocycline, a drug that suppresses microglia activation^47,49^. Taken together, it is unlikely that the cause of suppressed rebound sleep responses after sleep loss in IL-4Rα KO mice is due to inhibited microglial activation.

Our study has some limitations. In addition to macrophages, IL-4Rs are also expressed by other cell types, such as neurons^50,51^, astrocytes^52,53^, adipocytes^54^ and hepatocytes^55^. The function of IL-4 signaling in these cell types is unclear, but the possibility that deficient signaling in these cells contributes to the observed sleep phenotype cannot be completely discounted. For example, IL-4 elicits astrocytic expression of CNS growth factors, such as nerve growth factor^52,56^. The somnogenic effects of several tissue growth factors, including NGF, have been reported^57–59^. Further, our study does not provide a direct insight into the role of classically activated, M1, and resting macrophages in sleep signaling. Future studies are required to elucidate the role of dynamic changes in macrophage polarization in immune sleep signaling. We did not measure macrophage phenotypes; IL-4Rα KO mice have been widely used to study the consequences of M2 cell deficiency. There are more than a dozen studies (*e*.*g*.^18–21^) demonstrating that macrophages of IL-4Rα KO mice macrophages unable to go through the alternative activation pathway and the animals are M2 macrophage deficient. Further, we did not perform a rescue experiment, because specific enhancement of M2 cell population in the KO mice for a rescue experiment is not possible since the presence of IL-4Rα is necessary for M2 polarization. Theoretically, M2 polarization could be enhanced by targeting a downstream element in the IL-4 receptor-activated signal cascade. Unfortunately, these molecular tools for *in vivo* use are not yet available.

In summary, we described sleep deficiencies in an M2 macrophage-deficient mouse model. Sleep is not the only complex brain function in which M2 cells may play a role. IL-4 KO mice and T and B cell-deficient SCID mice have cognitive impairment in spatial learning tasks. Intriguingly, these learning deficiencies can be reversed by transplantation of IL-4–competent bone marrow or by the intravenous injection of M2, but not M1, macrophages^23,60^. Our findings are consistent with prior observations that normal macrophage number/activity is required for maintaining normal sleep after sleep loss and in moderately cold environment. They also add to the growing number of observations that peripheral signaling by the immune system and metabolic organs is key component of sleep regulation. Metabolic disorders and insufficient sleep are pressing public health issues in industrialized societies. Recent studies suggest a link between insufficient sleep or circadian disruption on the one hand and metabolic disorders on the other^61^. Identified cellular and biochemical links among sleep, metabolism and the immune system, such as macrophages, could potentially provide an accessible target for translational research and the development of effective therapies for sleep- and sleep loss-related metabolic disorders.

## Methods

### Animals

Breeding pairs of IL-4Rα KO (BALB/c-Il4ra^tm1Sz^/J) and Balb/cJ (control) mice were purchased from The Jackson Laboratories, Inc. and further bred at Washington State University for one generation. During the experiments, mice were housed in temperature controlled (30 ± 1 °C), sound attenuated isolation chambers on a 12:12-h light-dark cycle (lights on at 4 AM). The mice used in the experiments were first generation offspring of the original breeding pairs obtained from The Jackson Laboratories, Inc. Food and water were available *ad libitum* throughout all experiments. Animals were provided regular lab chow (Harlan Teklad, Product no. 2016), in which fats, proteins, and carbohydrates comprise 12%, 22%, and 66% of calories, respectively. All animal procedures were conducted in compliance with the recommendations in the Guide for the Care and Use of Laboratory Animals of the National Institutes of Health. All animal protocols were approved by the Institutional Animal Care and Use Committees at Washington State University.

### Surgery

At 4- to 6-months of age, male mice (IL-4Rα KO n = 11; Balb/cJ n = 11), were instrumented for sleep, locomotor activity, and body temperature recordings using ketamine-xylazine (87 and 13 mg/kg, respectively) anesthesia. For sleep recordings, three electroencephalographic (EEG) electrodes were implanted over the frontal and parietal cortices, and two electromyographic (EMG) electrodes were implanted within the nuchal muscles. All electrodes were connected to a plastic pedestal, which was secured to the skull with dental cement. Temperature-sensitive transmitters (MiniMitter telemetry system, STARR Life Sciences Corp., Oakmont, PA, USA) were implanted intraperitoneally for telemetry recordings. Mice were kept in individual cages and allowed 7–10 days to recover from surgical procedures and to acclimate to recording cables before baseline recordings were started.

### Sleep-Wake Recordings and Analysis

Cables were connected to the plastic pedestal to record EEG and EMG signals (digitized at 256 Hz). Cables were connected to commutators, which were routed to 12A5 amplifiers (Grass Model 15 Neurodata Amplifier System, Grass Instrument Division of Astor-Med, Inc., West Warwick, RI, USA). Signals were passed through an analog-to-digital converter and then recorded using the SleepWave software suite (Biosoft Studio, Hershey, PA). High- and low-pass filters were applied to EEG signals at 0.5 and 30.0 Hz, respectively; and to EMG signals at 100 and 10,000 Hz, respectively. Vigilance states including wakefulness (W), non-rapid-eye movement sleep (NREMS), and rapid-eye movement sleep (REMS) were determined off-line in 10-s epochs according to standard criteria, as described previously^34^. The duration of each vigilance state was calculated in 2-, and 12-h blocks. EEG data from artifact-free 10-s epochs was subjected to off-line spectral analysis via fast Fourier transformation to obtain EEG power data. Data in the range of 0.5 to 4.0 Hz during NREMS were used to compute EEG SWA. EEG SWA data from the baseline day was averaged across the 24-h recording period and used as a reference value. EEG SWA values for the baseline and experimental days were expressed as a percentage of this reference value “units”) in 2-h bins.

### Telemetry

Body temperature and locomotor activity were recorded using MiniMitter telemetry system and VitalView software. Average core body temperature and accumulated motor activity values were collected every 1 and 10 min, respectively, throughout the experiment, and averaged over 2-h bins.

### Experimental Procedures

#### Experiment 1: Spontaneous sleep, locomotor activity, and body temperature at thermoneutral ambient temperature (30 °C)

After a 7–10-day recovery and habituation period, spontaneous sleep-wake activity, locomotor activity, and body temperature data was collected for 72 h in IL-4Rα KO (n = 11) and WT (n = 11) mice. Recordings started at dark onset.

#### Experiment 2: Effects of sleep deprivation on sleep, locomotor activity, and body temperature in IL-4Rα KO mice

After baseline recordings, control (n = 9) and IL-4Rα KO (n = 9) mice were sleep deprived by gentle handling during the last 6 hours of the light phase. Sleep deprivation involved stimulation of animals by touching them with a soft paintbrush or gently tapping or shaking their cage. Minimal stimulation was applied, and stimuli were limited to circumstances in which animals displayed behavioral signs of drowsiness or sleep. The animals were allowed to eat and drink freely during sleep deprivation. Sleep-wake activity, body temperature, and locomotor activity were recorded immediately from the end of the sleep deprivation period for 24 h (recovery day), starting at dark onset. Two IL-4Rα KO and three control mice were excluded from data analyses due to artifacts in the EEG signals.

#### Experiment 3: Effects of cold exposure on sleep, locomotor activity, and body temperature in IL-4Rα KO mice

Two weeks after initial baseline recordings, another baseline was taken for 24 h at 30 °C ambient temperature in control (n = 8) and IL-4Rα KO (n = 9) mice. At dark onset, environmental temperature was reduced to 10 °C for 24 h. Sleep-wake activity, body temperature, and locomotor activity data were recorded throughout all experimental days.

#### Experiment 4: Effects of cold exposure on metabolic parameters

In a separate group of 3-month old male mice (IL-4Rα KO n = 8; Balb/cJ n = 8), oxygen consumption (VO_2_, ml/kg/h) was measured at 30 °C and 10 °C ambient temperatures via indirect calorimetry (Oxymax System, Columbus Instruments, Columbus, OH). Animals were habituated to cages for at least three days before the recordings.

#### Experiment 5: The effects of sleep deprivation on IL-4 expression in brown adipose tissue (BAT)

WT mice (n = 6) were sleep deprived for 6 h as described above. At the end of the sleep deprivation the mice were euthanized, and BATs removed and stored at −80 °C. Control mice were left undisturbed in their home cage and allowed to sleep *ad libitum* (n = 6); their BATs were removed at the same time as the sleep deprived animals’.

Quantitative real-time RT-PCR was used to analyze mRNA levels of IL-4. The tissue was homogenized, and total RNA was extracted by using TRIzol reagent according to the manufacturer’s protocol (Invitrogen, Carlsbad, CA, USA). RNA concentration was quantified by spectrophotometry at 260 nm. First-strand cDNA was synthesized using SuperScript III (Invitrogen). The PCR reaction mixture (20 μl) contained 4 μl of the diluted cDNA (20 ng total RNA), 10 μl of SsoAdvanced^TM^ SYBR Green Supermix (Bio-Rad, Hercules, CA, USA) and 0.4 μl of the primers at 10 μM. The primer sequences for IL-4 were (F) 5′ AGACGTCCTTACGGCAACAAG 3′ and (R) 5′ AGCACCCTGGAAGCCCTGC 3′ and for Cyclophilin A were (F) 5′ AAATGCTGGAC CAAACACAAA 3′ and (R) 5′ CTCATGCCTTCTTTCACCTTC 3′. The RT-reaction conditions were 30 s at 95 °C for enzyme activation followed by 40 cycles of 5 s at 95 °C and 30 s at 58 °C. Finally, a melting curve was generated by stepwise increasing temperature (0.5 °C increase every 5 s) for 60 cycles starting at 65 °C. If multiple peaks were observed during the melt curve analyses the data were not used. The reactions were performed in triplicate. Each reported cycle threshold (C_t_) value was an average of these values. Expression levels were analyzed using the ΔΔCt method. IL-4 transcript levels are normalized to the cyclophilin A levels.

### Statistics

Baseline NREMS, raid-eye movement sleep (REMS) and wakefulness (W) amounts, as well as the average body temperature and locomotor activity were calculated in 2-h blocks for control and IL-4Rα KO mice. Data were analyzed for normal distribution; no transformation was required prior to ANOVA. Two-way mixed analysis of variance (ANOVA) was performed to compare these metrics across a 24-h period (independent measure: genotype, repeated measure: time). When ANOVA indicated significant effects, Tukey’s HSD test was applied as *post hoc* analysis for all experiments. NREMS, REMS and W episode frequency and average episode durations were separately calculated for the 12-h dark and 12-h light periods; comparisons were made between experimental groups using Student’s t-tests.

To assess the effects of sleep deprivation, the amounts of NREMS, REMS and W, as well as EEG SWA and locomotor activity were calculated in 12-h blocks. Two-way mixed ANOVAs were performed across the dark period (independent measure: genotype, repeated measure: sleep deprivation).

To assess the effects of cold exposure, the amounts of NREMS, REMS and W episodes, as well as average body temperature during a 24-h baseline (30 °C) and a cold exposure (10 °C) day were calculated. Two-way mixed ANOVAs were performed to compare these metrics between baseline and cold exposure days in both experimental groups (independent measure: genotype, repeated measure: ambient temperature). An α-level of *P* < 0.05 was considered to be significant.

To assess VO_2_, values were averaged in 2-h blocks during the 24-h baseline (30 °C) and during cold exposure (10 °C). Three-way mixed ANOVA was performed across the 48-h recording period (independent measure: genotype, repeated measures: time and ambient temperature).

### Data Availability Statement

All relevant data are within the paper.
